# Incidence of cardiac arrest following implementation of a predictive analytics display in a pediatric intensive care unit

**DOI:** 10.1016/j.resplu.2024.100862

**Published:** 2025-01-02

**Authors:** Michael C. Spaeder, Laura Lee, Chelsea Miller, Jessica Keim-Malpass, William G. Harmon, Sherry L. Kausch

**Affiliations:** a Department of Pediatrics, University of Virginia School of Medicine, Box 800386, Charlottesville, VA 22908, USA; b Center for Advanced Medicine Analytics, University of Virginia School of Medicine, Box 800386, Charlottesville, VA 22908, USA

**Keywords:** Cardiac arrest, Intensive care units, Pediatric, Machine learning, Child, Infant

## Abstract

**Background:**

More than 90% of in-hospital cardiac arrests involving children occur in an intensive care unit (ICU) with less than half surviving to discharge. We sought to assess the association of the display of risk scores of cardiovascular and respiratory instability with the incidence of cardiac arrest in a pediatric ICU.

**Methods:**

Employing supervised machine learning, we previously developed predictive models of cardiovascular and respiratory instability, incorporating real-time physiologic and laboratory data, to display risk scores for potentially catastrophic clinical events in the subsequent 12 h. Clinical implementation with risk scores displayed on large screen monitors in multiple areas throughout the ICU was finalized in July 2022. We compared the incidence of cardiac arrest events in the 18-months pre- and post-implementation.

**Results:**

The cardiac arrest incidence rate dropped from 3.0 events (95% CI 2.0–4.4) to 2.4 events (95% CI 1.6–3.5) per 1000 patient days following implementation. We observed a 50% increase in the rate of cardiac arrest events where return of spontaneous circulation (ROSC) was achieved (*p* = 0.025). The incidence rate of cardiac arrest without ROSC dropped from 1.4 events (95% CI 0.7–2.4) to 0.4 events (95% CI 0.1–0.9) per 1000 patient days (incidence rate difference = 1.0 (95% CI 0.13–1.87), *p* = 0.01).

**Conclusions:**

We observed a non-significant decrease in the rates of cardiac arrest events and an increase in the rate of cardiac arrests events where ROSC was achieved following the implementation of a predictive analytics display of risk scores.

## Introduction

Approximately 40 children experience in-hospital cardiac arrest requiring cardiopulmonary resuscitation (CPR) each day in the United States, with less than half surviving to discharge^1^, ^2^. Early recognition of patients at risk for cardiac arrest may provide clinicians with the opportunity to intervene, potentially preventing cardiac arrest altogether^3^.

The primary focus for years in pediatric cardiac arrest research centered on the care of the child experiencing, or following, cardiac arrest. More recently, there has been considerable attention paid toward cardiac arrest prevention^3^, ^4^, ^5^, ^6^. One of the central tenets of cardiac arrest prevention is the fostering of situational awareness amongst front line clinical staff for patients at elevated risk for clinical deterioration^3^, ^4^, ^5^, ^6^. Concurrent with this work in cardiac arrest prevention, there has an increased adoption of clinical decision support tools in the pediatric intensive care unit (PICU), including predictive analytics, aimed at identifying patients at risk for clinical outcomes such as cardiac arrest^7^, ^8^, ^9^, ^10^, ^11^.

Employing supervised machine learning, we previously developed predictive models of cardiovascular and respiratory instability, incorporating real-time physiologic and laboratory data, to display risk scores for the development of sepsis and respiratory failure in the subsequent 12 hours^12^, ^13^. Our objective was to assess the association of the display of risk scores for potentially these catastrophic clinical events with the incidence of cardiac arrest in a combined cardiac and medical/surgical PICU.

## Materials and methods

We performed a pre-post observational study of cardiac arrest events in the PICU at the UVA Health Children’s Hospital from January 2021 to June 2024. We included all admissions to the PICU, a 24-bed combined cardiac and medical/surgical PICU. The Institutional Review Board at the University of Virginia School of Medicine approved this study (IRB-HSR #231514). We followed the Strengthening the Reporting of Observational Studies in Epidemiology guidelines^14^.

We previously trained and validated random forest models for the development of cardiovascular and respiratory instability in the subsequent 12 h in our PICU^12^, ^13^. Model features include: (1) continuous cardiorespiratory waveforms (3 leads of ECG sampled at 240 Hz, pulse plethysmography at 120 Hz, and invasive blood pressure tracings at 120 Hz); (2) vital signs (heart rate, respiratory rate, peripheral oxygen saturation, invasive blood pressure, and sample-and-hold noninvasive blood pressure) sampled at 0.5 Hz; (3) laboratory values (serum sodium, potassium, chloride, bicarbonate, blood urea nitrogen, creatinine, glucose, calcium, white blood cell count, hematocrit, platelet count); (4) patient age; and (5) vital sign calculations (e.g., heart rate variability, cross-correlation of peripheral oxygen saturation and respiratory rate). Full model details are available in the source literature.

**Fig. 1:**
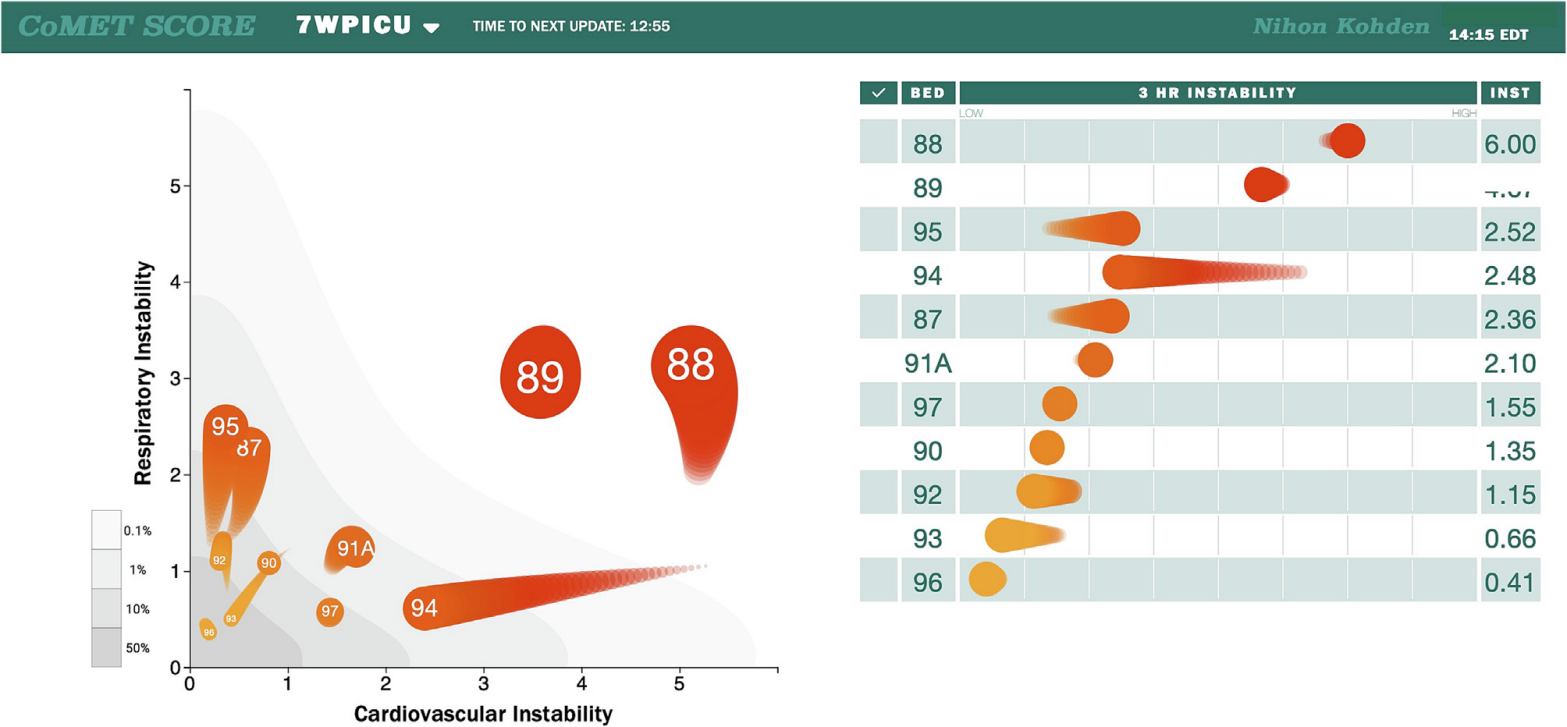
**Continuous Monitoring of Event Trajectories (CoMET) display.** Left: Risk estimates, or CoMET scores, are displayed for both the cardiovascular (*x*-axis) and respiratory (*y*-axis) instability domains and represent the fold increase in risk of an event in the next 12 h. A score of 1 means that the risk of an event is at the average risk for the pediatric intensive care unit, with higher numbers meaning higher risk. The bed number is displayed in the head of the CoMET and the tail represents score trajectory over the previous 3 h. For example, the patient in room 88 is at high risk for cardiovascular instability and has experienced an increase in risk for respiratory instability over the last 3 h. Right: A composite score of joint risk equal to the square root of the sum of squared cardiovascular and respiratory instability scores is displayed, ranking patients in the pediatric intensive care unit from highest to lowest risk score by room number. The 3-hour tail of the CoMET is also shown to demonstrate the degree of recent stability or instability.

Model risk estimates are calculated every 15 min and displayed using the Continuous Monitoring of Event Trajectories (CoMET®) (Nihon Kohden Digital Health Solutions, Irvine, CA) predictive analytics tool (Fig. 1). Risk estimates, or CoMET scores, are displayed for both the cardiovascular and respiratory instability domains and represent the fold increase in risk as compared to average risk for an event to occur in the subsequent 12 h. Additionally, a composite score of joint risk equal to the square root of the sum of squared cardiovascular and respiratory instability scores is displayed, ranking patients in the PICU from highest to lowest risk score by room number.

The PICU at the UVA Health Children’s Hospital is comprised of two adjacent 12-bed units (PICU West and PICU North). A large screen monitor, positioned at the main work station (nursing, respiratory therapy, and advanced practice provider/physician) for each unit, displays CoMET scores for the corresponding unit. Additionally, reciprocal monitoring for the adjacent unit is displayed on a separate monitor at both main work stations. A web-based version of the display is available on all computer workstations in the PICU, including bedside computers, and can be accessed on personal digital devices. The average hourly CoMET scores are populated within the electronic health record clinical overview flowsheet after validation by the bedside nurse. The clinical response protocol dictates that an advanced practice provider or physician be alerted and evaluate patients with CoMET scores that have increased by 2 over the previous three hours (e.g., 1.5–3.6) or for a CoMET score greater than 4.

Implementation of the CoMET predictive analytics tool was finalized in July 2022. Prior to go-live, all PICU staff (nurses, physicians, advanced practice providers, respiratory therapists) completed computer-based learning modules. Virtual and in-person education sessions were conducted in the timeframe surrounding implementation and on a recurring basis following implementation. Response protocols are posted at all nursing stations as well as at each bedside computer.

Review of the electronic patient record and an internal quality improvement database was conducted to identify all cardiac arrest events occurring in the PICU. Post-hoc, we defined a cardiac arrest event as any episode of CPR with chest compressions lasting a minimum of 60 s. To focus on the predictive ability of the display, we excluded events that occurred within one hour of PICU admission. Additionally, we determined if the cardiac arrest event was associated with return of spontaneous circulation (ROSC) as well as the use of extracorporeal life support, with or without ROSC prior to cannulation. To better characterize the realities of prolonged resuscitations in which sustained ROSC is not maintained, multiple events within a two-hour window were treated as a single event. We defined case fatality using the previously reported definition by Lasa of failure to rescue following cardiac arrest as death in the PICU^5^.

We designated the 18-months prior to implementation as the pre-CoMET period (1/22–6/22) and following a 3-month washout period, designated the subsequent 18-months as the post-CoMET period (10/22–3/24).

Distribution of continuous variables was assessed using the Wilk-Shapiro test for normality. Continuous variables were compared using Student’s T test or Wilcoxon rank sum testing as appropriate. Categorical variables were compared using chi-square test or Fisher’s exact testing as appropriate. To compare incidence rate differences with person-time denominators between time periods we employed a statistical test based on the normal approximation for the binomial distribution as previously described^15^. To characterize incidence rates over time, statistical process control charts (U-charts) were constructed^3^, ^4^. We performed multilevel mixed effects logistic regression modeling with patient as a random effect to account for multiple observations from individual patients. Type I error was set at 0.05. All calculations were performed using StataBE 18.0 (STATA Corporation, College Station, TX).

## Results

There were 8,658 patient days during the pre-CoMET period and 10,409 patient days during the post-CoMET period. We included 51 cardiac arrest events during the period of study, 26 events in the pre-CoMET period and 25 events in the post-CoMET period. The median patient age at the time of cardiac arrest event was 10 months (interquartile range (IQR) 1 month to 4 years) with no difference between time periods. Cardiac arrest events were more common in patients with primary cardiac disease (80% of events) with no difference between time periods.

**Fig. 2:**
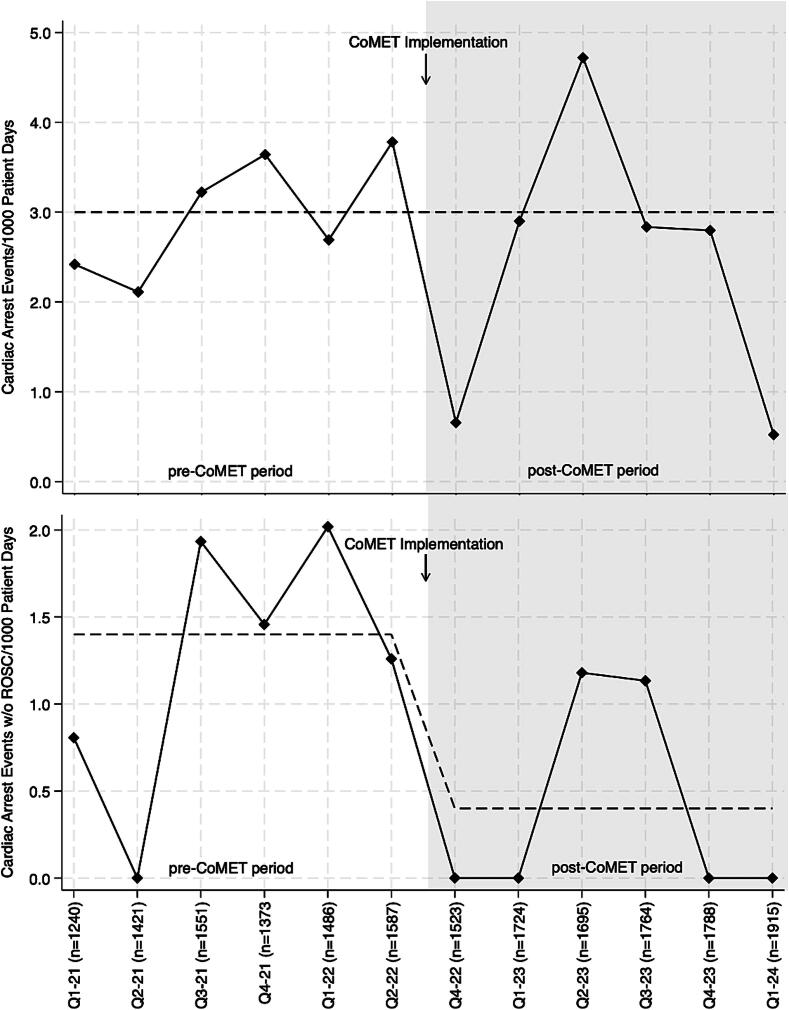
Statistical process control charts (U-chart) for the incidence rates of cardiac arrest events (top) and cardiac arrest events without return of spontaneous circulation (bottom) by quarter (*n* = patient days each quarter). ROSC, return of spontaneous circulation. Note: Q3 of 2022 was washout period.

The cardiac arrest incidence rate dropped from 3.0 events per 1000 patient days (95% CI 2.0–4.4) in the pre-CoMET period to 2.4 events per 1000 patient days (95% CI 1.6–3.5) in the post-CoMET period (Fig. 2). In the post-CoMET period, we observed a 50% increase in the rate of cardiac arrest events where ROSC was achieved (*p* = 0.025). Thus, the incidence rate of cardiac arrest without ROSC dropped from 1.4 events per 1000 patient days (95% CI 0.7–2.4) in the pre-CoMET period to 0.4 events per 1000 patient days (95% CI 0.1–0.9) in the post-CoMET period (incidence rate difference = 1.0 (95% CI 0.13–1.8)) (Fig. 2). Employing patient days from 2023, the incidence rate difference equates to 7 (95% confidence interval 1–13) fewer cardiac arrest events per year in the post-CoMET period where ROSC was not achieved. Among events where ROSC was achieved, there was no difference in the duration of time prior to ROSC between the time periods (8 (IQR 3–13) versus 5 (IQR 4–10) minutes).

We did not observe a difference in the rates of patients receiving event-associated extracorporeal life support, with or without ROSC prior to cannulation, between time periods. Case fatality was 42.5% and did not differ between time periods. Twenty-seven percent of events were associated with tracheal intubation, and there was no difference between time periods. We did observe an increase in the rate of ROSC with tracheal intubation-associated events from 50% in the pre-CoMET timeframe to 88% following implementation of CoMET.

## Discussion

More than 90% of pediatric in-hospital cardiac arrests occur in an intensive care unit^2^. Cardiac arrest prevention remains a priority in the PICU though reports of effective interventions remain rare. Dewan and colleagues reported a 52% decrease in CPR events after implementation of a quality improvement initiative involving several time-intensive interventions including twice daily safety huddles and bedside mitigation signs^3^. The development of predictive analytics which incorporate real-time physiologic and laboratory data into risk estimates holds promise for identification of high-risk patients without unduly burdening practitioner workload.

Implementation of predictive models of clinical instability, incorporating real-time physiologic and biochemical data, may aid clinical personnel in identifying patients at risk for cardiac arrest, allowing for earlier and more targeted intervention. A recent scoping review of supervised machine learning prediction models in pediatric critical care identified a small number of models trained and validated on the outcome of cardiac arrest^8^. Only a small subset of these models incorporated the use of real-time physiologic and laboratory data in deriving risk estimates and to our knowledge, none are currently implemented in a clinical setting^8^, ^16^, ^17^, ^18^, ^19^. Multicenter efforts to train and validate models specifically on the outcome of cardiac arrest are needed.

Training and validating models on rare events such as pediatric in-hospital cardiac arrest can be difficult as it requires a sufficient number of events to develop meaningful models. The models that we have incorporated into CoMET were trained and validated on the outcomes of sepsis (cardiovascular instability) and emergency intubation (respiratory instability)^12^, ^13^. We surmise that there is sufficient overlap in the physiology observed in the patient at risk for cardiac arrest as seen during the early stages of sepsis and septic shock and impending respiratory failure. Early proactive action and clinical intervention may lead to less fulminant cardiac arrest events as well as increased vigilance and preparedness for high-risk procedures (e.g., intubation) and these clinical actions may have contributed to the improved rate of ROSC.

**Fig. 3:**
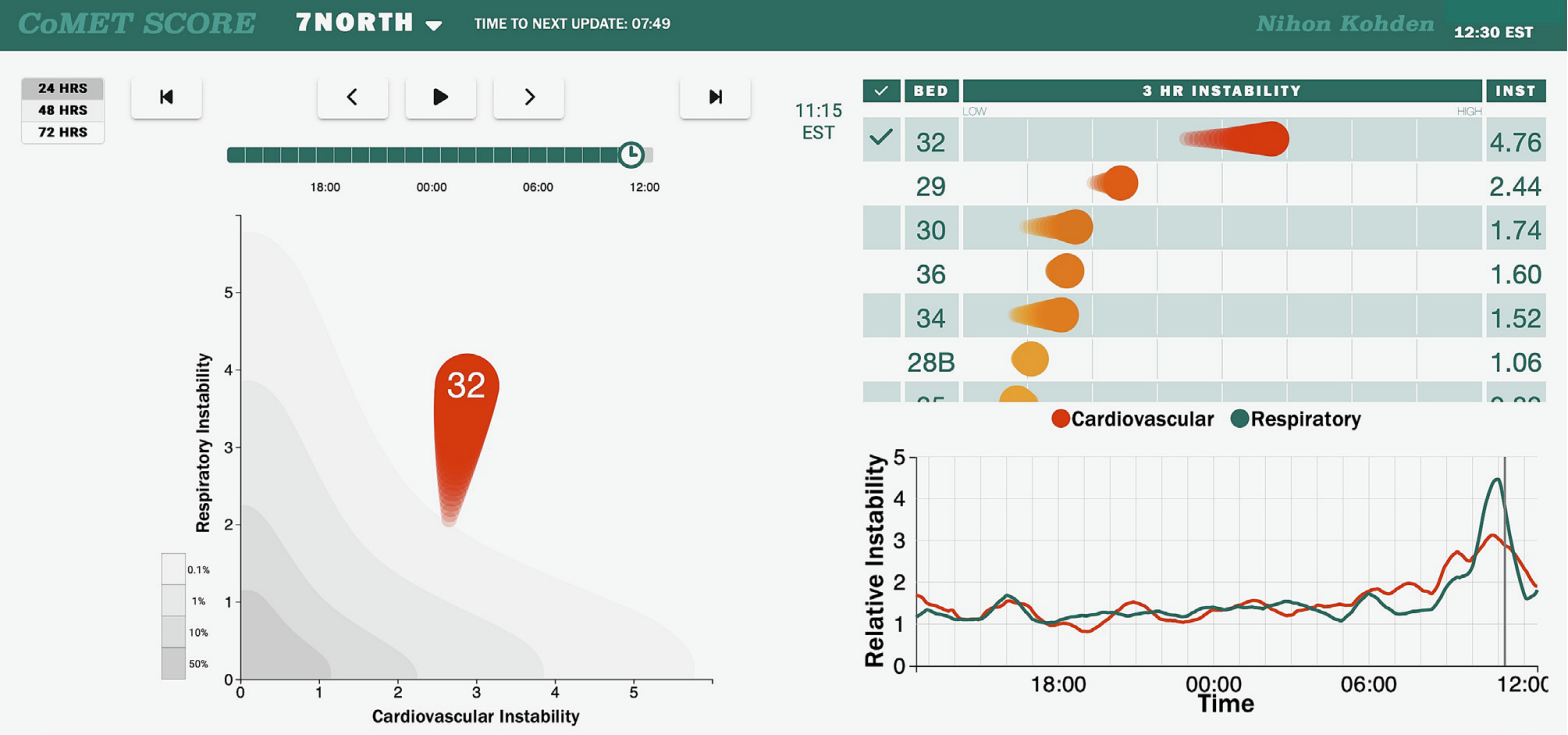
**Continuous Monitoring of Event Trajectories (CoMET) case example.** Case presentation of a 4-month infant with rapidly progressive respiratory failure on the morning after abdominal surgery leading to intubation and subsequent cardiac arrest with return of spontaneous circulation. Right: Individual patients can be selected for review by selecting the box to the left of their bed number. This brings up the graph shown in the lower right, which is a 24-, 48-, or 72-hour graph showing the progress of the patient’s CoMET scores over a defined period, with cardiovascular instability as the red line and respiratory instability as the green line. Note the rise in CoMET scores starting around 9 am with the subsequent intubation event occurring around 11 am. Left: The patient’s CoMET is large and bright red at the time of intubation, emphasizing the current high level of risk, mainly in respect of their risk of respiratory instability. The tail of the CoMET demonstrates that this instability has substantially progressed over the prior 3 h. (For interpretation of the references to colour in this figure legend, the reader is referred to the web version of this article.)

Nishisaki and colleagues recently reported a secondary analysis of the ICU-RESUS clinical trial and ancillary CPR-NOVA study which demonstrated that 15% of all cardiac arrests occurring in the PICU were associated with tracheal intubations^20^. We observed a rate nearly double though we hypothesize that this reflects the heavy representation of patients with primary cardiac disease in our cohort. While we did not discern a significant decrease in the proportion of cardiac arrest events associated with tracheal intubation in the post-CoMET period, we did observe a 76% increase in the rate of these case achieving ROSC following CoMET implementation. The CoMET display for a representative patient is presented in Fig. 3 to illustrate how CoMET may have aided in the early identification of impending respiratory failure and preparedness for high-risk intubation.

There are several limitations that should be noted. This was a single-center academic PICU with a high proportion of cardiac diagnoses, so the population may not represent all centers. Due to how data was obtained retrospectively, we do not have access to the type of cardiac arrests (e.g., pulseless electrical activity, ventricular tachycardia). While we adjusted for multiple events in individual patients, we did not adjust for other potential confounders such as type of cardiac arrest, illness category, or age. Furthermore, we were not able to objectively characterize process and balancing metrics such as regarding protocol adherence and alert burden. Over the period of study, 4.7% of the time one or both risk scores exceeded 4, equating to 21 instances per day. The random forest predictive models were developed using our internal cohort, employing cross-validation without a separate validation cohort. We are in the initial stages of performing a multicenter external validation of these models as well as investigating associations with other adverse outcomes. Additionally, this work was designed as a beginning point to characterize pragmatic effectiveness and we acknowledge that future work focusing on implementation and workload impacts are necessary for long-term use and adoption.

## Conclusions

We observed a non-significant decrease in the rates of cardiac arrest events and an increase in the rate of cardiac arrests events where ROSC was achieved following the implementation of a predictive analytics display of risk scores.

## Funding and support

The University of Virginia School of Medicine maintains a research collaboration agreement with Nihon Kohden Digital Health Solutions which provides complimentary access and support to the Continuous Monitoring of Event Trajectories (CoMET®) software.

## CRediT authorship contribution statement

**Michael C. Spaeder:** Writing – original draft, Visualization, Supervision, Project administration, Methodology, Investigation, Formal analysis, Conceptualization. **Laura Lee:** Writing – review & editing, Investigation, Data curation. **Chelsea Miller:** Writing – review & editing, Investigation. **Jessica Keim-Malpass:** Writing – review & editing, Methodology, Conceptualization. **William G. Harmon:** Writing – review & editing, Methodology, Conceptualization. **Sherry L. Kausch:** Writing – review & editing, Project administration, Methodology, Investigation, Formal analysis, Data curation, Conceptualization.

## Declaration of competing interest

The authors declare that they have no known competing financial interests or personal relationships that could have appeared to influence the work reported in this paper.
